# Quantitative Analysis of Vitreo-Macular Adhesion in Diabetic Patients Without Proliferative Retinopathy

**DOI:** 10.7759/cureus.109467

**Published:** 2026-05-22

**Authors:** Priyanka, David Aggarwal, Pradeep Venkatesh, Mihika Dube, Brijesh Takkar, Bhavana Sharma

**Affiliations:** 1 Ophthalmology, All India Institute of Medical Sciences, Bhopal, Bhopal, IND; 2 Ophthalmology, Aggarwal Clear Sight Eye Hospital, Patiala, IND; 3 Ophthalmology, Dr. RP Centre for Ophthalmic Sciences, All India Institute of Medical Sciences, New Delhi, New Delhi, IND; 4 Ophthalmology, Ram Krishna Dharmarth Foundation (RKDF) Medical College and Hospital, Bhopal, IND; 5 Ophthalmology, Smt Kanuri Santhamma Center for Vitreoretinal Diseases, LV Prasad Eye Institute, Hyderabad, IND

**Keywords:** diabetic macular edema, diabetic retinopathy, optical coherence tomography, posterior vitreous detachment, vitreomacular adhesion

## Abstract

Background: Vitreomacular adhesion (VMA) is a physiological stage of posterior vitreous detachment that plays a significant role in the progression of diabetic retinopathy. This study aims to quantitatively assess the percentage of VMA in diabetic patients without proliferative disease compared to non-diabetic controls.

Methods: This is a prospective, double-masked, cross-sectional study conducted at a tertiary eye care center in central India. Consecutive diabetic patients were evaluated. Proliferative diabetic retinopathy (PDR) was excluded. Optical coherence tomography (OCT) of the macula was done using four radial scans (rotational angulation set at 45°, 90°, 135°, and 180°). Patients were classified as either VMA+ or VMA. A quantitative analysis was performed for each case to calculate the percentage length of the retinal surface with attached vitreous cortex (%VMA).

Results: Sixty-eight eyes of 40 patients and 50 eyes of 25 age-matched controls were included for analysis. Mean duration of diabetes mellitus (DM) was 7.9 +/-5.4 yrs. 65/68 eyes (96%) of DM group were VMA+, while 42/50 eyes (84%) of the control group were VMA+ (p=0.032), OR=4.12 (CI= 1.03-16.45). Percentage VMA (%VMA) was higher for the DM group at all meridians, but comparison with controls did not reveal statistically significant findings (average %VMA 65% vs. 58%, p=0.251). Though statistically insignificant, the average %VMA was higher for patients with non-proliferative diabetic retinopathy (NPDR; 71%), in comparison to patients without diabetic retinopathy (DR; 63.4%) or with macular edema (61%) (p=0.554).

Conclusion: We could not establish a statistically significant quantitative variation of %VMA between diabetic and non-diabetic individuals, or amongst early manifestations of DR. Future studies in advanced stages of DR with upcoming OCT systems are needed to confirm our findings.

## Introduction

Diabetic retinopathy (DR) is a well-described sight-threatening complication of diabetes. Worldwide, 93 million people have DR, among which 17 million people have proliferative diabetic retinopathy (PDR), 21 million have diabetic macular edema (DME), and 28 million have vision-threatening DR [1]. DR is characterized by different stages, including preretinal adhesions that may lead to tractional retinal detachment [2,3].

The strongest points of attachment of the vitreoretinal interface are present at the macula, optic nerve, near the ora serrata, and around the blood vessels. Weakening of the adhesion between the posterior vitreous cortex and the internal limiting membrane (ILM), along with vitreous body liquefaction, results in posterior vitreous detachment (PVD). Anomalous vitreoretinal interface represents an abnormal separation of the vitreous cortex from the ILM. Partial detachment of the vitreous in the perifoveal area with intact retinal contour represents vitreomacular adhesion (VMA) and is an early stage of vitreoretinal separation [4]. Vitreomacular interface (VMI) has a key role in many macular disorders, including DR [4]. Traditionally, VMA is known to be common in cases with DR, although vitreous detachment is quicker to occur in patients with diabetes mellitus (DM) without DR. The prevalence of VMI diseases has been reported as 7% to 16% in eyes with DME, and yearly incidence as 4.5% [5,6]. VMI anomalies range from VMA, epiretinal membrane (ERM), to vitreomacular traction (VMT). VMA is considered a possible risk factor for the development of DR in patients with type 2 DM and influences the therapeutic outcomes. The role of VMA and the ERM in affecting treatment outcomes of patients with DME has been reported previously; however, there are very few reports in the literature stating the role of VMA in DM patients without retinopathy [7].

Optical coherence tomography (OCT) is a routinely employed tool in research and therapeutics pertaining to DR and is more sensitive in detecting vitreomacular abnormalities than conventional techniques [8]. The previous studies on VMI in DR are mostly retrospective studies with major limitations. These studies have included eyes treated with laser or intravitreal injections, and eyes that had undergone surgery, which may alter the course of VMI. Most of these studies are qualitative and have been done using a single OCT scan of the macula and have not evaluated eyes without retinopathy or with early retinopathy. The current study compares VMA in DM patients with or without DR quantitatively using multiple radial OCT scans through the macula. Further, a comparative analysis between no DR, non-proliferative DR (NPDR), and diabetic macular edema (DME) for VMA is also presented.

## Materials and methods

This is a prospective, double-masked, cross-sectional study conducted at a tertiary eye care center in central India. The study was approved by the Institutional Human Ethics Committee (LOP/2018/IM0195, dated July 11, 2018). Informed consent was obtained from all patients, and the study was in accordance with the tenets of the Declaration of Helsinki and its later amendments. 

Consecutive patients with type 2 DM presenting between August 2018 and January 2019 were evaluated. These patients had presented to the outpatient department, retina clinic, or diabetic retinopathy screening clinic of the institute. Patients with ocular disease, apart from cataract, or any media opacity precluding examination, and those with PDR were excluded. PDR was excluded as the OCT calculations described later are not possible in patients with tractional retinal detachment, hemorrhage, or neovascular fronds. Patients with any history of ocular treatment, including that for cataract, high myopia, and DR, were also excluded. Patients having systemic disease apart from hypertension, for example, hyperlipidemia and nephropathy, were also excluded. Age-matched non-diabetic controls were included from the outpatient department, and exclusion criteria were similar to those for cases. All serological investigations had been done within one month of inclusion in the study. Selected patients underwent a complete ocular evaluation. Recorded data included age, gender, duration of DM, and associated systemic hypertension. Fundus evaluation of all patients was done under mydriasis by a single retina surgeon, who noted the presence of complete posterior vitreous detachment and classified DR as the presence of NPDR or DME. PDR was ruled out with fluorescein angiography when in doubt clinically.

All OCT imaging was done by a single technician using a single OCT machine (HDOCT-Cirrus, Carl-Zeiss Meditec, Dublin, CA, USA). This machine has an in-built protocol which allows high definition B-scans with a longitudinal resolution better than 10 microns. It also allows the operator to set an angle for the B scan based on the point of fixation. Four radial scans were obtained for each patient with the rotational angulation set at 45°, 90°, 135°, and 180°. OCT scans with signal strength less than 7/10 were repeated, and if signal strength remained low, the eyes were excluded from analysis. These images were then graded by two independent graders blinded from each other’s findings using standard criteria and classified into either VMA+ or VMA-. VMA positivity (VMA+) was defined based on the International Vitreomacular Traction Study (IVTS) Group classification: an elevation of the posterior vitreous cortex from the retinal surface in the perifoveal region, with persistent, continuous attachment of the vitreous within a 6-mm radius of the fovea, without any structural changes to the underlying retinal layers or foveal contour [9]. In contrast, VMA- was designated if there was either complete posterior vitreous detachment (PVD) within the macular area or no evidence of vitreous separation. Discrepancies between the two graders were resolved through open consensus discussion with a third senior retina specialist. Presence of epiretinal membranes (ERMs) and vitreoschisis was specifically noted. Further, a quantitative analysis was performed for each case to calculate the percentage of the length of the retinal surface with attached vitreous cortex (%VMA). These measurements were done using the callipers available within the software. Measurements were obtained separately for each angle of the B scan, and the average percentage for each eye was also obtained. Thus, the objectives of this study were: (i) (primary objective) to quantitatively evaluate and compare the percentage length of the retinal surface with attached vitreous cortex (%VMA) using multi-meridian radial OCT scans in diabetic patients without proliferative disease against age-matched non-diabetic controls; (ii) (secondary objective) to perform an intra-group comparative analysis among diabetic individuals to determine if the %VMA correlates with the severity of early-stage diabetic retinopathy.

Data was serially entered in a Microsoft Excel (Microsoft Corporation, Redmond, WA, USA) worksheet, and statistical analysis was performed with IBM SPSS Statistics for Windows, version 20 (IBM Corp., Armonk, NY, USA). For analysis, patients with DM were evaluated against the controls without DM for %VMA. Further, patients with DM were grouped as having no DR, NPDR, or DME, and a comparison was also done among these groups. An independent t-test was used for comparison between two groups, whereas one-way analysis of variance (ANOVA) was used to compare non-parametric variables of three groups. Post hoc Tukey’s honest significant difference (HSD) test was applied for testing inter-group differences, wherever ANOVA was used. Chi-square test was used for analyzing parametric variables, and Mantel-Haenszel estimate was used for evaluating the unadjusted odds ratio (OR). Correlation coefficients were calculated using Pearson’s coefficient for correlation. Only a two-tailed p value < 0.05 was considered statistically significant, and all confidence intervals (CI) described are true for 95% of the sample.

## Results

Sixty-eight eyes of 40 patients and 50 eyes of 25 controls were included for analysis. The mean age of diabetic patients was 56.85 +/- 8.57 yrs, while it was 57.0 +/-5.48 yrs for controls (p=0.916). Thirty-six (53%) of the eyes of the DM group were right eyes, while 25 (50%) of the control eyes were right eyes (p=0.752). Eighteen (45%) of the DM patients were female patients, while 14 (56%) of the controls were female patients (p=0.337). The overall mean duration of DM was 7.9 +/-5.4 yrs. 65 (96%) eyes of the DM group were VMA+, while 42 (84%) eyes of the control group were VMA+ (p=0.032). Thus, VMA was significantly more common in the DM group qualitatively, which had an OR of 4.12 (CI= 1.03-16.45, p= 0.044). ERMs were seen in four (5.88%) of DM patients, and none of the controls. Vitreoschisis was noted in five (7.35%) of DM patients and one (2%) of the controls. Complete posterior vitreous detachment (PVD; on clinical examination) was seen in two (2.94%) eyes of the DM group and four (8%) eyes of the control group. Hypertension was significantly more common in the DM group in comparison to the control group (34 (50%) vs. 12 (24%), p=0.004). The comparison between these groups has been summarized in Table 1.

**Table 1:** Comparison between cases and controls Data are presented as mean ± standard deviation (SD) for continuous variables and number (n) with percentage (%) for categorical variables. Comparisons between diabetic and control groups were performed using the independent samples t-test for continuous variables and the Chi-square test or Fisher’s exact test for categorical variables, as appropriate. Corresponding test statistics (t-values and χ² values) are reported along with p-values. A p-value < 0.05 was considered statistically significant. VMA: vitreo-macular adhesion; PVD: posterior vitreous detachment

Characteristic	Diabetic eyes (n=68)	Control eyes (n=50)	Test statistic	P value
Mean age (yrs)	56.85 +/- 8.57	57.0 +/-5.48	t = 0.113	0.916
Male/female ratio	22 (55%)/18 (45%)	11 (44%)/14 (56%)	χ² = 0.922	0.337
Right/left eyes	36 (53%)/32 (47%)	1 (50%)/1 (50%)	χ² = 0.100	0.752
VMA+	65 (95%)	42 (84%)	χ² = 4.577	0.032
Epiretinal membrane	4 (5.88%)	0 (0%)	Fisher’s exact	0.136
Vitreoschisis	5 (7.35%)	1 (2%)	Fisher’s exact	0.240
Complete PVD clinically	2 (2.94%)	4 (8%)	Fisher’s exact	0.399
Hypertension	34 (50%)	12 (24%)	χ² = 8.189	0.004
%VMA @ 45°	64.6 +/- 29.5	58.9 +/-37.8	t = 0.920	0.365
%VMA @ 90°	63.4 +/- 29.2	56.8 +/- 37.8	t = 1.070	0.292
%VMA @ 135°	62.03 +/- 27.9	56.7 +/- 37.7	t = 0.883	0.385
%VMA @ 180°	69.5 +/-27.4	60.4 +/- 38.6	t = 1.498	0.137
Average %VMA	64.9 +/- 27.8	57.9 +/- 37.2	t = 1.170	0.251

DM eyes were compared with control eyes for %VMA at all the angulations. The %VMA was found to be maximum in the horizontal meridian (180°) in both groups, while it was the least at 135°. Data has been summarized in Table 1. Though %VMA was always higher for the DM group, comparison did not reveal a statistically significant finding in any meridian.

Within the diabetic group, 39 (57.3%) eyes had no retinopathy, 17 (25%) eyes had NPDR, and 12 (17.6%) eyes had DME. Amongst patients with NPDR, moderate retinopathy was most common 10 (59%), and only three (18%) cases had more than severe retinopathy. Details have been presented in Table 2. These groups were similar in terms of age, duration of DM, and male/female and right/left eyes ratios. Hypertension was much more common in DME (10, 83%) than NPDR (nine, 53%) or no DR (15, 38%) (p=0.024). These groups were similar in the presence of VMA or clinically detectable PVD. ERMs were seen most commonly in DME (one, 8.33%) and vitreoschisis in the no retinopathy (four, 10.25%) group.

Comparison was done amongst the DM patients for the association of %VMA with the level of retinopathy (Table 2).

**Table 2:** Comparison amongst DM patients Data are presented as mean ± SD for continuous variables and number (n) with percentage (%) for categorical variables. Comparisons among the three groups were performed using one-way analysis of variance (ANOVA) for continuous variables and the Chi-square test or Fisher’s exact test for categorical variables. Corresponding test statistics (F-values and χ² values) are reported along with p-values. A p-value < 0.05 was considered statistically significant. DM: diabetes mellitus; DR: diabetic retinopathy; NPDR: non-proliferative diabetic retinopathy; DME: diabetic macular edema; VMA: vitreo-macular adhesion; PVD: posterior vitreous detachment

Characteristic	No DR (n=39)	NPDR (n=17)	DME (n=12)	Test statistic	P value
Mean age (yrs)	57.27+/-0.57	59.41+/-8.89	52.0+/-8.12	F = 2.835	0.066
Duration of DM (yrs)	7.68+/-17.84	10.8+/-11.55	10.67+/-10.12	F = 0.877	0.421
Male/female ratio	19 (48.7%)/20 (51.3%)	11 (64.7%)/6 (35.3%)	6 (50%)/6 (50%)	χ² = 1.265	0.531
Right/left eyes	20 (51.3%)/19 (48.7%)	9 (52.9%)/8 (47.1%)	7 (58.3%)/5 (41.7%)	χ² = 0.183	0.912
VMA+	36 (92.3%)	17 (100 %)	12 (100 %)	χ² = 2.334	0.311
Epiretinal membrane	3 (7.69%)	0 (0%)	1 (8.33%)	Fisher’s exact	0.491
Vitreoschisis	4 (10.25%)	1 (5.88%)	0 (0%)	Fisher’s exact	0.475
Complete PVD clinically	2 (5.1%)	0 (0%)	0 (0%)	Fisher’s exact	0.465
Hypertension	15 (38.5%)	9 (52.9%)	10 (83.3%)	χ² = 7.469	0.024
%VMA @ 45°	62.95+/-15.67	70.41+/-29.88	61.60+/-28.65	F = 0.454	0.637
%VMA @ 90°	61.29+/-32.88	71.48+/-29.11	58.56+/-28.62	F = 0.924	0.402
%VMA @ 135°	60.85+/-40.02	66.71+/-29.01	59.09+/-26.99	F = 0.332	0.719
%VMA @ 180°	68.64+/-43.99	75.58+/-27.88	63.87+/-26.32	F = 0.684	0.508
Average %VMA	63.42+/-46.31	71.07+/-28.50	60.78+/-26.86	F = 0.596	0.554

Similar to the overall findings, %VMA was seen to be maximum in the horizontal meridian (180^◦^). Though VMA was found to be highest for the NPDR group in all meridians, the difference between the three groups was not found to be statistically significant (p>0.05 in all comparisons). This finding was re-ascertained with post hoc analysis, whereupon application of the honestly significant difference (HSD) test for inter-group comparison, no significant difference was found for any variable tested between any combination of the three groups.

The correlation between age and %VMA was found to be R=-0.32 in controls (p=0.019), while it was found to be much weaker (R=-0.03) in DM patients (p=0.781). Amongst the DM patients, the overall correlation between duration of DM and average %VMA was found to be very poor (R=0.18, p=0.135). Similarly, the correlation between duration of DM and %VMA was found to be very poor for patients with no DR (R=0.28, p=0.079), NPDR (R=-0.04, p=0.870), and DME (R=0.30, p=0.340).

To remove any confounding impact of hypertension, the data were re-analyzed after excluding patients with hypertension. DM patients (n=34, mean %VMA=64.65%) and controls (n=38, mean %VMA=58.65) were compared for the difference in %VMA, but as before, the difference was found to be insignificant (p=0.471). Similarly, for DM patients, groups with no DR (n= 24, mean %VMA=62.40%), NPDR (n=8, mean %VMA=74.64%), and DME (n=2, mean %VMA=52.53%) were compared for %VMA, but results were found to be statistically insignificant (p=0.610).

## Discussion

Our prospective study is better than many previous qualitative studies in excluding systemic illnesses, excluding treated and pseudophakic patients, using multiple fovea-centred OCT scans, including clinical findings on VMI and DR status, and including controls [10-18]. Though %VMA was found to be slightly higher in DM patients than controls, and in the NPDR patients amongst the DM patients, we could not prove our findings to be statistically significant. To our knowledge, quantitative and controlled analysis for VMA in DM and DME are lacking in the literature.

DM has been shown to have subclinical effects on retinal function with electrophysiology, even before the clinical appearance of retinopathy. Such changes have been termed “diabetic neuroretinopathy” [10]. On similar lines, we had planned our study to decipher pre-clinical VMI changes in diabetics by including patients both with and without retinopathy, in effect looking for diabetic “vitreo-retinopathy”. Mikhail et al. studied 100 patients and found previous cataract surgery and previous laser therapy for DR to have a very strong impact on VMI alterations, showing the necessity of maintaining stringent exclusion criteria while planning OCT studies in DR or DM [11]. Most previous studies on VMI changes in DM, however, have been done qualitatively in DME only, and that too with very weak exclusion criteria. This is perhaps due to interest in the visual effect of VMI changes, as well as their impact on treatment with anti-VEGF agents. A notable study has been done by Wong et al., who found the presence of ERMs to be associated with poor response to treatment, and VMA and spontaneously releasing VMA to be associated with good response to therapy [12]. On the same lines, Sadiq et al reported a beneficial effect of VMA+ at baseline on the treatment of DME eyes with anti-VEGF therapy in a post hoc analysis of the Ranibizumab for Edema of the Macula in Diabetes (READ)-3 trial [12]. Khan et al. studied 198 patients undergoing macular laser for central DME with OCT, and found “partial vitreous separation” (12%) and ERMs (14%) to be present frequently [14]. Similarly, in 2006, Kim et al., while describing the types of DME with time domain OCT, mentioned more than 15% to have “hyaloid traction” [15]. Kulikov et al. studied 105 eyes with DME and found VMI anomalies of any kind (including VMA) to lead to poor visual outcomes following therapy [16]. Ophir et al. analyzed 186 eyes with DME and retinal traction in 2010, and found central foveal traction to be more common than non-central traction [17]. Chang et al. studied 180 patients with DME and found an association of VMI anomalies with poor visual outcomes and a thicker central retinal thickness [5]. Ghazi et al. studied 48 eyes with persistent DME and found a high prevalence of VMI anomalies (27.1% ERM, 16.7% VMA, and 8.3% both) in persistent DME, documenting the superiority of OCT in detecting such abnormalities [8]. Recently, Adhi et al. studied 110 eyes with diabetic retinopathy using 3D swept source OCT and noted thick posterior hyaloid and vitreoschisis more in DME as compared to NPDR [18]. Gaucher et al. studied 98 eyes in diabetic patients with and without DME and noted a high prevalence of peri-foveolar PVD with foveolar attachment in DME [19]. Some of these studies have been summarized in Table 3.

**Table 3:** Comparison with previous qualitative studies VMA: vitreo-macular adhesion, DR: diabetic retinopathy, NPDR: non-proliferative DR, DME: diabetic macular edema, PDR: proliferative DR, ERM: epiretinal membrane, VMIA: vitreo-macular interface anomalies, PRP: pan-retinal photocoagulation, VEGF: vascular endothelial growth factor

Study	Place, year	Sample size (eyes/patient)	Design	Findings	Remark
Kim et al. [15]	USA, 2006	164/119	Retrospective observational case series	Posterior hyaloid tension in 15.6%	Not controlled
Ophir et al. [17]	Israel, 2010	186/122	Retrospective study	Vitreo-foveal traction in 13.4%, extra-foveal traction in 10.8%	Study focused on tractional changes and not adhesion as such
Chang et al. [5]	Taiwan, 2012	244/180	Retrospective observational study	Up to 6.6% had anomalous VMA	Not controlled, included patients with cataract surgery, macular laser, and PRP
Khan et al. [14]	UK, 2015	198/198	Retrospective, observational	Partial vitreomacular separation in 12%	Not controlled, included patient with laser, and PDR
Adhi et al. [18]	USA, 2016	110/67	Prospective cross-sectional	Thick posterior hyaloid noted in 0% of no DR, 19% of NPDR, 55% of PDR, 41% of DME. Vitreoschisis in 45% of no DR, 48% of NPDR, 65% of PDR, 63% of DME	Included patient with PDR, anti-VEGF, and laser. Used vitreous windowing method
Wong et al. [12]	UK, 2017	104/77	Prospective, observational	43% had central ERM, 63% had eccentric ERM	Not controlled, focused
Mikhail et al. [11]	UK, 2018	146/100	Retrospective, observational	VMIA in 18.5%	Not controlled, included patient with cataract surgery, and laser
Present study	India, 2019	118/65	Prospective, cross-sectional	VMA noted in 92.3% of no DR, 100% of NPDR, and DME	Quantitative analysis with multiple radial scans, with very good exclusion criteria

For these reasons, VMI has been envisaged as a target of enzymatic vitreolysis, and different therapies have been hypothesized for the management of DME [14,20,21].

In contrast, literature is lacking with regard to the impact of DM on VMI in patients without DR or with NPDR. Normally, collagen fibres in conjunction with high molecular weight proteins and glycosaminoglycans (like laminin, fibronectin, and chondroitin) are essential in fusing the posterior vitreous cortex with the inner retinal surface. In DM patients, the vascular dysfunction ensues inflammatory cascades whose end product is believed to be fibrosis and a “sticky” posterior vitreous cortex [22]. In other words, VMA is increased. Nasrallah et al clinically evaluated DM eyes with DME or DR but no DME in 1988 for PVD [23]. They stated the vitreous cortex to be detached more commonly in eyes without DME. Foos et al. evaluated diabetic vitreous gel in 1980 for the presence of PVD in autopsied specimens and concluded PVD to be more common in diabetics without proliferative retinopathy [24]. Gella et al. analyzed the diabetic VMI using ultrasound and found incomplete PVD to be present in > 75% of the diabetic eyes studied [25]. Further, they also concluded incomplete PVD to be associated with advanced stages of retinopathy. The overall rates for PVD, however, were similar to those for the general population (63%) [25]. A recent study in 2017 by Nesmith et al. included 141 DM patients and performed a 25-line raster scan over the fovea [26]. The authors found the area of VMA to decline with age and with increasing PVD. The study was, however, limited by not accounting for lens status and not including patients with DME [26]. 

We have avoided drawing any conclusion on the clinical presence of PVD, as only six eyes in our study sample had it overall, and the numbers are too few. OCT is a better modality to evaluate VMA than clinical, autopsy, or ultrasound studies mentioned prior because of its much higher resolution [23-25]. Though comparing our OCT findings with these studies may not be correct, it is possible that our findings on %VMA are due to a higher number of cases with no or minimal retinopathy, as only three cases had more than severe NPDR, while only 12 had recent onset DME, and we had excluded PDR. From our data, it seems %VMA is similar in cases with no DR and “early” DR. The studies mentioned in Table 3 have been done in cases of DR with visual loss that required treatment and have documented extensive qualitative changes at the VMI. It is possible that VMA increases in association with established sight-threatening DR, which would be in contrast to diabetic “neuro-retinopathy” that precedes clinical changes [9]. With advancing retinopathy or in cases where some treatment of DR has been done, statistically significant changes may be more apparent while using our methodology, as has also been seen in the study by Gella et al. [25].

Limitations

Due to our stringent methodology, we could enroll only 68 eyes in the diabetic group, and the number of patients with NPDR and DME was less compared to those with no DR. In the NPDR subgroup, most cases had mild-moderate retinopathy. This could be a reason for some results regarding NPDR and DME getting masked statistically.

This is the first analysis of its kind, and further studies should be done on %VMA in DM and DR, before we conclude whether VMA alone can be the target of therapy or a prognostic marker for DR or DME. This is because a “non-parametric %VMA” will be a more effective study tool than simply classifying as VMA+ or VMA- qualitatively. We have used SD-OCT in this study, and with the advent of new imaging methods designed specifically for the mobile vitreous, findings shall get more accurate in the future [18]. Also, we have evaluated only 6 mm of the retinal surface; application of wide-field OCT will give a better estimate of %VMA, as VMA is known to go through stages and vary at different levels of the retinal periphery [27-29].

## Conclusions

We analyzed %VMA for its variations in DM patients, and in eyes with early stages of DR. We could not ascertain a statistically significant variation of %VMA between diabetic and non-diabetic individuals, or amongst the early manifestations of DR. It is possible that advanced DR may show larger differences in %VMA and, as this is the first analysis on% VMA in DM, our findings should be confirmed by future studies before clinical relevance is interpreted.
